# Exploring Coronavirus Disease 2019 Risk Factors: A Text Network Analysis Approach

**DOI:** 10.3390/jcm14062084

**Published:** 2025-03-19

**Authors:** Min-Ah Kang, Soo-Kyoung Lee

**Affiliations:** 1 Department of Nursing, Keimyung College University, Daegu 42601, Republic of Korea; makang@kmcu.ac.kr; 2 Department of Medical Informatics, College of Nursing & Health, Kongju National University, Kongju 32588, Republic of Korea

**Keywords:** coronavirus disease 2019, text network analysis, risk factors

## Abstract

**Background/Objectives:** The coronavirus disease 2019 (COVID-19) pandemic has significantly affected global health, economies, and societies, necessitating a deeper understanding of the factors influencing its spread and severity. **Methods:** This study employed text network analysis to examine relationships among various risk factors associated with severe COVID-19. Analyzing a dataset of published studies from January 2020 to December 2021, this study identifies key determinants, including age, hypertension, and pre-existing health conditions, while uncovering their interconnections. **Results:** The analysis reveals five thematic clusters: biomedical, occupational, demographic, behavioral, and complication-related factors. Temporal trend analysis reveals distinct shifts in research focus over time. In early 2020, studies primarily addressed immediate clinical characteristics and acute complications of COVID-19. By mid-2021, research increasingly emphasized long COVID, highlighting its prolonged symptoms and impact on quality of life. Concurrently, vaccine efficacy became a dominant topic, with studies assessing protection rates against emerging viral variants, such as Alpha, Delta, and Omicron. This evolving landscape underscores the dynamic nature of COVID-19 research and the adaptation of public health strategies accordingly. **Conclusions:** These findings offer valuable insights for targeted public health interventions, emphasizing the need for tailored strategies to mitigate severe outcomes in high-risk groups. This study demonstrates the potential of text network analysis as a robust tool for synthesizing complex datasets and informing evidence-based decision-making in pandemic preparedness and response.

## 1. Introduction

The coronavirus disease 2019 (COVID-19) pandemic has significantly impacted global health systems, economies, and societies. While the acute phase of the pandemic has passed, COVID-19 continues to pose significant health challenges, including the emergence of new variants, long-term complications (long COVID), and mental health effects [1,2]. These ongoing concerns underscore the necessity of continued research on risk factors associated with COVID-19 severity and outcomes. As of December 2021, the long-term effects of COVID-19 and its impact on various populations remain insufficiently understood, necessitating further investigation.

COVID-19′s high transmissibility and variable severity have driven extensive research into its biological, environmental, and social determinants. Early studies highlighted age, comorbidities, and socioeconomic status as key risk factors [2]. However, while these studies provided valuable insights, they often relied on traditional epidemiological models that struggled to capture the evolving and interconnected nature of these risks. Additionally, prior studies often examined risk factors in isolation, lacking a holistic perspective on how different factors interact within the broader research landscape.

The evolving characteristics of COVID-19 have revealed significant disparities in outcomes based on demographic features such as age, sex, race, and socioeconomic status. Studies have shown that older adults and individuals with pre-existing conditions face higher risks of severe illness, while marginalized communities experience disproportionate effects due to limited healthcare access and economic constraints [3]. Variations in health policies, preventive measures, and vaccine distribution add to the complexity of global responses [4]. Addressing these challenges requires advanced analytical approaches that synthesize vast amounts of data to reveal underlying patterns across diverse datasets.

To address these challenges, researchers increasingly turn to computational and data-driven approaches [5]. Advances in data analytics and computational modeling have enabled new approaches, particularly text network analysis, which has emerged as an effective method for identifying patterns and associations within textual data. Unlike traditional epidemiological studies that rely on structured datasets, text network analysis systematically explores unstructured textual information. This methodology is particularly well-suited for addressing the research gap in understanding the relationships among various COVID-19 risk factors, as it enables the detection of hidden connections and emerging trends within vast literature [6].

TNA differs from other computational methods, such as machine learning and natural language processing (NLP), in several ways. Machine learning techniques typically require labeled datasets for supervised training or employ unsupervised clustering algorithms to categorize data, often necessitating large-scale training datasets. NLP, on the other hand, is focused on processing and extracting meaning from text but may lack the ability to systematically map relationships between key concepts over time. In contrast, TNA constructs networks of semantic relationships between key terms, allowing researchers to visualize knowledge structures, detect thematic shifts, and track the evolution of scientific discourse [7].

Traditional epidemiological models primarily rely on structured datasets and predefined variables, which may limit their ability to capture complex interconnections and dynamic research trends. In contrast, TNA enables the systematic exploration of unstructured textual data, allowing researchers to uncover emerging themes, detect hidden relationships, and track the evolution of scientific discourse over time. Unlike static epidemiological models, TNA can continuously adapt to new research developments and identify unexpected associations between risk factors [8].

A key advantage of TNA is its ability to integrate diverse information sources, including academic literature, social media, and public health reports, to uncover meaningful relationships and emerging trends [9]. For example, during the early stages of the pandemic, social media served as a critical channel for disseminating public health information yet also contributed to the spread of misinformation. TNA enables researchers to systematically analyze and contextualize such data, distinguishing between reliable findings and misleading narratives [10].

Previous studies have often examined COVID-19 risk factors in isolation, lacking a holistic perspective on how different determinants interact within the broader research landscape. Additionally, many studies struggled to capture dynamic research shifts, as they relied on static datasets. By employing TNA, this study provides a comprehensive, data-driven synthesis of risk factor interactions over time, bridging these gaps and offering new insights into the evolving research landscape.

Recognizing the importance of understanding COVID-19 risk factors and their interconnections, this study employs text network analysis to investigate relationships among various disease-associated determinants. Specifically, the study aims (1) to identify key keywords in studies related to risk factors for severe COVID-19, (2) to analyze the relationships among keywords and classify subgroups based on network clusters, and (3) to examine the temporal research trends in studies related to severe COVID-19 risk factors.

Through this approach, this study seeks to provide a comprehensive and data-driven synthesis of risk factor interactions over time. By leveraging text network analysis, this research aims to bridge gaps in existing knowledge, offering insights that can inform public health strategies, policy decisions, and pandemic preparedness efforts.

## 2. Materials and Methods

This quantitative content analysis used text network analysis to extract key keywords related to severe COVID-19 risk factors, classify network-based subgroups, and identify research trends over time.

### 2.1. Search Strategy

**Figure 1:**
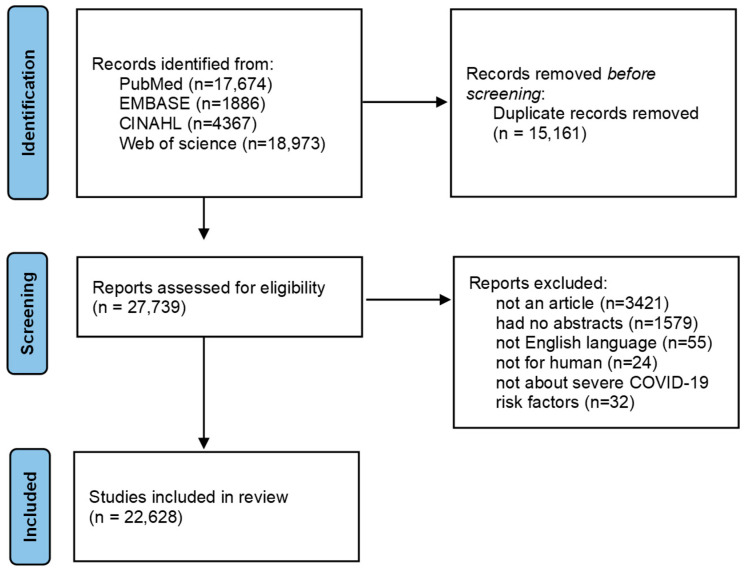
PRISMA flow diagram of article selection process.

Keywords for data collection were designed using a combination of natural language and MeSH (Medical Subject Heading) terms. Keywords included ‘COVID-19’, ‘SARS-CoV-2’, ‘2019-nCoV’, ‘risk’, ‘risk factor’, ‘severe’, ‘critical’, ‘severity’, ‘intensive care unit’, ‘respiratory failure’, and ‘fatal’ (Table A1). A literature search was conducted across four major academic databases: PubMed, Scopus, Web of Science, and EMBASE, to ensure comprehensive coverage of peer-reviewed studies. Boolean operators (“AND” and “OR”) were applied to construct the search queries. Specifically, “OR” was used to combine synonyms and related terms (e.g., “COVID-19” OR “SARS-CoV-2” OR “2019-nCoV”), while “AND” was used to refine searches by including key concepts (e.g., “COVID-19” AND “risk factor” AND “severe”). The final search queries were tailored to the syntax of each database. The search was limited to articles published in English and indexed in peer-reviewed journals, with a publication period from January 2020 to December 2021. To ensure transparency, a PRISMA flow diagram (Figure 1) was included to illustrate the article selection process.

### 2.2. Inclusion and Exclusion Criteria

A total of 42,900 articles were retrieved from the databases. After removing 15,161 duplicates, 27,739 articles were screened based on their titles and abstracts. Inclusion criteria were as follows: (1) articles published between January 2020 and December 2021; (2) studies on COVID-19 risk factors related to disease severity and transmission; (3) peer-reviewed publications in English; and (4) articles providing relevant epidemiological or clinical data. Exclusion criteria were as follows: (1) non-peer-reviewed papers, preprints, and opinions; (2) articles without abstracts or full-text availability; (3) studies involving non-human subjects (e.g., mice, cats, dogs, gorillas); and (4) research focusing on unrelated topics such as MERS or information security. Two independent reviewers, a researcher and a nursing professor, screened the articles and ultimately selected 22,628 articles for data analysis.

### 2.3. Data Extraction and Analysis

Data extraction focused on keyword identification, cleaning, statistical validation, and visualization. The following steps were performed:

(1) Keyword Extraction and Cleaning
  - Keywords were extracted from the titles, abstracts, and keywords of the selected articles using Python 3.9, Natural Language Toolkit (NLTK) 3.6.7, and Text Rank algorithm.
  - Standardization was applied to account for variations in capitalization, pluralization, abbreviations, and special characters.
  - Synonyms were consolidated into single representative terms, ensuring consistency across the dataset.
(2) Keyword Analysis and Visualization
  - Key keywords were analyzed using term frequency analysis and Term Frequency-Inverse Document Frequency (TF-IDF) with Python’s Sklearn module (Scikit-learn 0.24.2).
  - Results were visualized using Gephi 0.9.2, a widely used open-source tool for network analysis.
(3) Clustering and Network Classification
  - Clusters were identified using modularity-based algorithms and PageRank centrality measures in Gephi.
  - Nodes with low similarity were filtered to improve clustering accuracy.
  - The Jaccard similarity coefficient threshold was set at 0.065 to exclude weak associations. The threshold (0.065) was determined based on both heuristic analysis and empirical validation. Following prior research [11], network visualization was used to assess optimal threshold selection. Additionally, multiple threshold values (0.05, 0.06, 0.07) were tested to balance network density and meaningful keyword relationships. The threshold of 0.065 was selected as it minimized noise while retaining key associations.
(4) Temporal Trend Analysis
  - Temporal trends were analyzed by computing monthly similarity indices based on frequency matrices and Jaccard coefficients.
  - Cut-off points were determined by identifying peaks and troughs in similarity trends using statistical methods, including moving averages and Z-scores, to ensure objective detection.
  - Four distinct time intervals were identified, highlighting emerging keywords in each period.

This methodology provides a systematic approach to understanding the evolving landscape of severe COVID-19 risk factors, ensuring a data-driven framework for public health insights and policymaking.

## 3. Results

### 3.1. Characteristics of Severe COVID-19 Risk Factor Studies and Key Keywords

**Figure 2:**
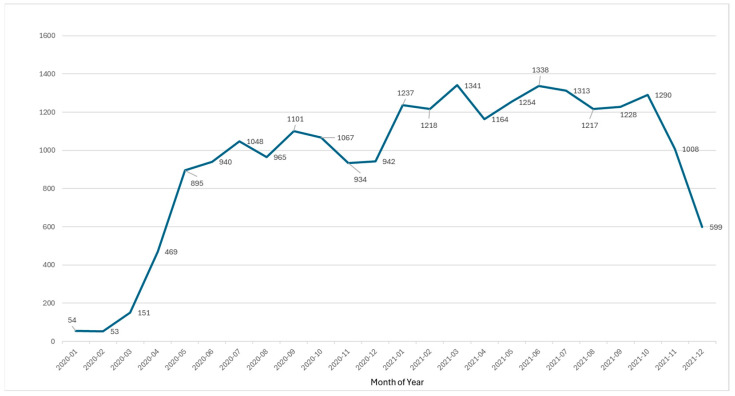
Monthly publications of severe COVID-19 risk factor studies.

The collected studies on severe COVID-19 risk factors included articles published in international and domestic journals between January 2020 and December 2021. A total of 22,628 studies were analyzed. Monthly publication trends showed a rapid increase, starting with 54 studies in January 2020 and peaking at 895 in May 2020. Approximately 1000 studies were published monthly until October 2021, after which the number decreased (Figure 2).

The surge in early 2020 likely reflects the global research response to COVID-19. The peak in May 2020 may correspond to the initial wave of studies focusing on early risk-factor identification and pandemic mitigation strategies. After October 2021, the decline in publications likely resulted from a research shift toward vaccine efficacy, long COVID, and post-pandemic recovery efforts. Figure 2 illustrates these trends, highlighting the evolving research priorities over time in response to global health events and policy changes.

**Figure 3:**
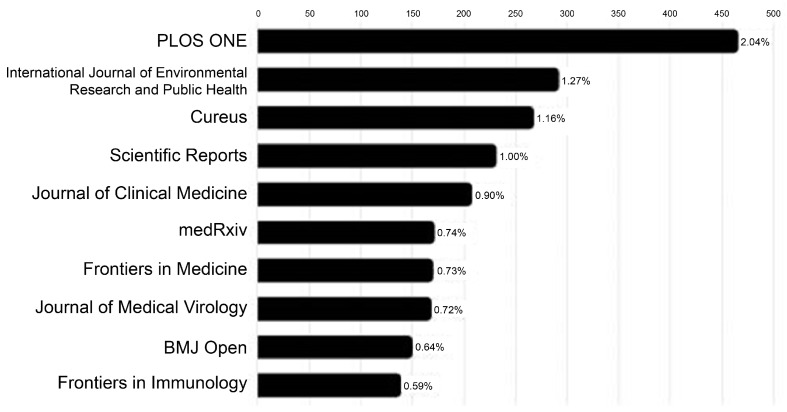
Journal distribution of search articles.

The retrieved studies were published in 5410 journals, with the top 15–20 journals accounting for approximately 9.8% of all articles (Figure 3). The top 10 journals included PLOS ONE (2.04%), International Journal of Environmental Research and Public Health (1.27%), Cureus (1.16%), Scientific Reports (1.00%), and Journal of Clinical Medicine (0.90%).

Table 1 presents keyword frequency and PageRank centrality values. Frequently appearing keywords included ‘age’ (3224 times), ‘treatment’ (2934), ‘diabetes’ (1504), ‘hypertension’ (1230), and ‘obesity’ (1045). PageRank centrality identified influential keywords such as ‘non-invasive ventilation’, ‘IgG’, ‘hyperglycemia’, ‘hypertension’, and ‘diabetes.’ Keywords with both high frequency and centrality included ‘hypertension’, ‘diabetes’, and ‘age. A detailed list of keywords is provided in Table A2.

### 3.2. Network Analysis of Keywords

**Figure A1:**
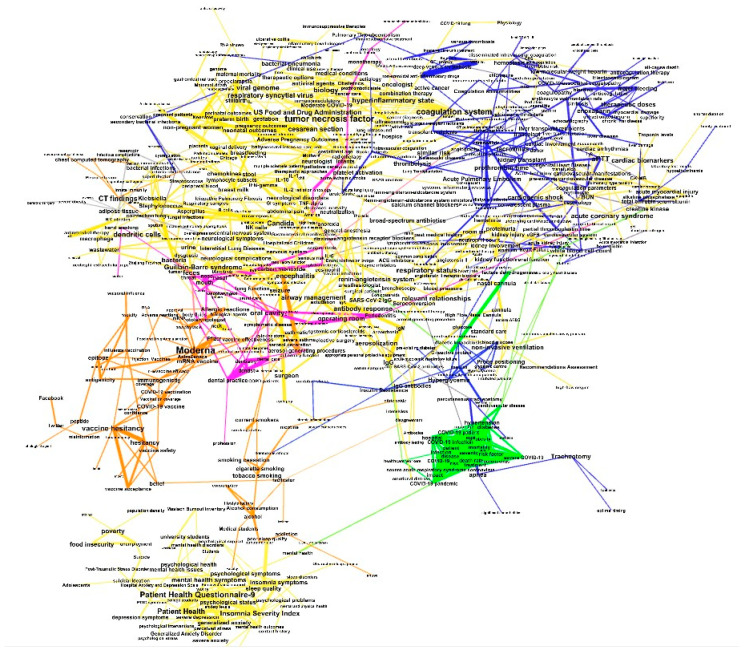
Spring network map of severe COVID-19 risk factors.

To examine structural trends in studies on severe COVID-19 risk factors, a text network analysis was conducted to visualize the knowledge structure. Using a PageRank algorithm analysis, 1492 keywords were identified. Applying a Jaccard similarity coefficient ≥0.065, the dataset was filtered to 346 nodes and 672 links, removing keywords with low frequency or weak connectivity. The final selected keywords represent core risk factors for severe COVID-19. Figure A1 presents a spring map visualization of the network.

**Figure A2:**
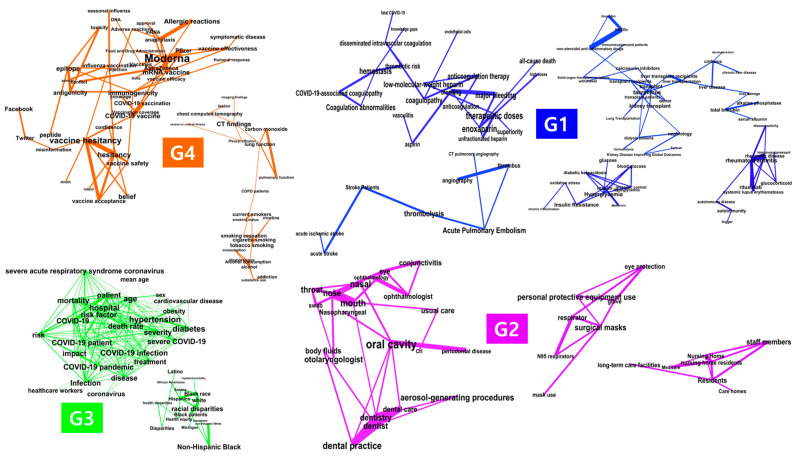
Clustering of severe COVID-19 risk factors: Groups 1–4.

A total of 21 clusters were identified and categorized into five major thematic groups: (1) biomedical, (2) occupational and environmental, (3) demographic, (4) health behavior, and (5) complication factors (Figure A2).

These clusters reflect key aspects of severe COVID-19 risk factors. For example, the biomedical cluster includes terms such as “cytokine storm” and “coagulopathy”, which are closely associated with severe disease progression. The demographic cluster highlights age-related risks, particularly among elderly populations. Similarly, the occupational and environmental cluster encompasses COVID-19 exposure risks in work environments.

While this cluster includes terms such as mouth and dentists, these are strongly linked to occupational hazards in healthcare settings. Studies have consistently identified dentists and other frontline workers as high-risk groups due to their frequent exposure to aerosol-generating procedures (AGPs). This underscores the importance of infection control measures and personal protective equipment (PPE) usage in these work environments.

**Figure A3:**
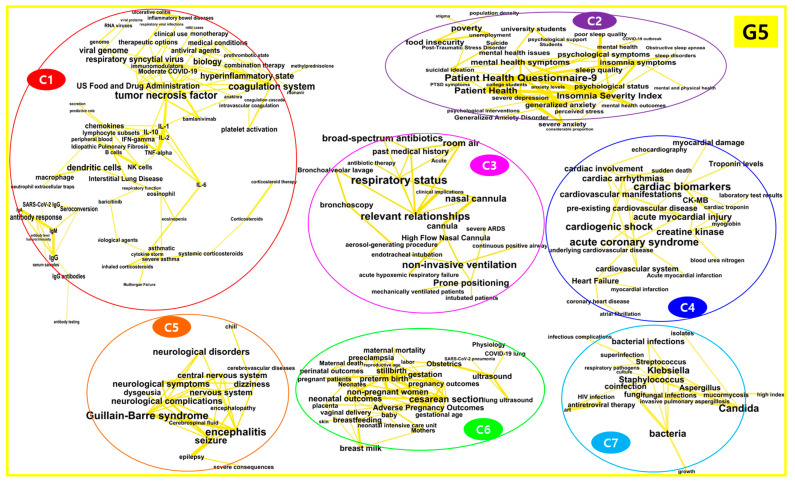
Group 5: Complication factors.

As shown in Figure A3, Group 5 (complication factors) was further divided into seven subcategories based on complications affecting organ systems (e.g., cardiovascular, respiratory, and immune responses). The keywords for each group are summarized in Table 2, illustrating that the major risk factors for severe COVID-19 are multifaceted and interrelated. The clustering results provide a structured overview of the interconnections between various risk factors, offering insights into how different domains contribute to severe COVID-19 outcomes. These findings highlight the multifaceted nature of COVID-19 risk and the necessity for interdisciplinary approaches in pandemic research.

### 3.3. Temporal Trends of Severe COVID-19 Risk Factor Studies

**Figure 4:**
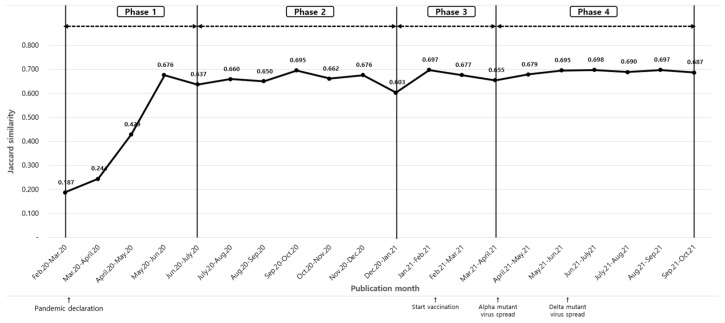
Phase setting by Jaccard similarity.

Temporal trends were analyzed using frequency matrices and Jaccard similarity coefficients to identify keyword usage changes over time. Keywords were examined monthly for 24 months and segmented into four phases. Phase 1 (February–June 2020) included keywords appearing ≥10 times with ≥100 total occurrences. Phase 2 (July–December 2020) included keywords with ≥10 occurrences and ≥50 total frequency. Phase 3 (January–March 2021) identified emerging keywords with ≥5 occurrences. Phase 4 (April–September 2021) highlighted keywords appearing ≥10 times with ≥20 total frequency (Figure 4).

Table 3 shows keyword frequencies by phase, including terms such as ‘obesity’, ‘social distancing’, ‘dyspnea’, ‘psychological well-being’, ‘long COVID’, and ‘mRNA vaccine’. Detailed frequencies are in Table A3.

## 4. Discussion

### 4.1. Principal Findings

The rapid spread of COVID-19 in early 2020 led to an exponential rise in research on transmission, mortality, and risk factors. Identifying contributors to severe COVID-19 became a global priority, informing public health interventions and guiding future research. This study employed text network analysis to examine key concepts and structural relationships in studies published between January 2020 and December 2021, providing insights into the evolving knowledge landscape.

Our findings identified ‘age’ as the most frequently mentioned keyword, aligning with prior studies indicating that older adults are more vulnerable due to weakened immune function and chronic inflammation [12]. ‘Hypertension’ emerged as a central keyword with both high frequency and PageRank centrality, confirming its role as a predictor of severe outcomes [13]. These findings highlight the need for targeted interventions for high-risk groups, particularly older adults and patients with pre-existing conditions. Additionally, recent research suggests that metabolic risk factors, such as obesity, diabetes, and metabolic syndrome, present a greater risk for severe complications than individual diseases alone [14]. This underscores the importance of a multidimensional approach to addressing complex risk factors. Some studies suggest that metabolic factors act as independent risk factors for severe COVID-19, while others indicate that their impact is mediated through hypertension and systemic inflammation [15]. This suggests the need for further research using causal modeling techniques to disentangle these interrelated pathways.

‘Non-invasive ventilation (NIV)’ exhibited high PageRank centrality despite lower frequency, indicating strong connections to other risk factors. However, many studies analyzing NIV use were conducted in hospital settings, which could introduce selection bias. The frequency of NIV use in severe COVID-19 cases may reflect institutional protocols rather than universal best practices [16]. Further investigation is needed to determine the optimal use of NIV across different patient populations and healthcare settings.

### 4.2. Network and Cluster Analysis

Network analysis identified five primary thematic clusters: biomedical, occupational, demographic, behavioral, and complication-related factors. The biomedical cluster featured terms such as ‘coagulopathy’, ‘hyperglycemia’, and ‘rheumatoid arthritis’, reinforcing the significance of underlying medical conditions. Consistent with previous research, coagulopathy was associated with increased mortality risk, highlighting the need for close monitoring of coagulation markers [17].

Occupational and environmental clusters focused on healthcare workers and long-term care facilities, underscoring occupational risks related to aerosol-generating procedures and inadequate protective equipment [18]. These findings support enhanced workplace protections for healthcare workers and vulnerable populations.

The demographic cluster emphasized ‘age’, ‘obesity’, and ‘race’, reflecting disparities in COVID-19 outcomes. Higher infection and mortality rates among Black and Hispanic populations have been widely documented, but the underlying causes of these disparities are complex [19]. Such disparities may arise from a combination of biological factors, socioeconomic conditions, and healthcare accessibility issues. Understanding the root causes of these disparities is crucial for developing equitable public health interventions. To effectively reduce racial and ethnic health disparities, a multi-faceted approach is required, which includes the following: Reducing socioeconomic inequalities through policies aimed at improving financial security, employment opportunities, and housing conditions. Enhancing healthcare access by addressing systemic inequities in insurance coverage and expanding medical services in underserved communities. Eliminating cultural and linguistic barriers to ensure equitable communication of health information and medical guidance. By implementing these strategies, public health initiatives can more effectively mitigate COVID-19 disparities and promote long-term health equity.

Behavioral clusters included ‘vaccine hesitancy’ and ‘alcohol consumption’, underscoring modifiable risk factors. Vaccine hesitancy remains a major barrier to herd immunity and outbreak control [19,20,21], necessitating public health campaigns to improve vaccine acceptance. Additionally, lifestyle factors such as smoking and alcohol consumption compromise immune function, increasing the risk of severe COVID-19. Smokers are 2–14 times more likely to develop severe cases than non-smokers, reinforcing the need for smoking cessation and alcohol reduction programs [22,23,24,25].

### 4.3. Inter-Cluster Relationships

This study highlights the interconnected nature of different clusters. Biomedical factors such as hypertension and obesity were closely linked to behavioral risk factors, including diet and physical inactivity. Similarly, occupational exposure correlated with demographic factors, as older adults and minority groups were more likely to work in high-risk frontline positions. Understanding these inter-cluster relationships is crucial for designing holistic public health strategies that address multiple interacting risk factors rather than isolated variables.

This study underscores the importance of a comprehensive approach to mitigating severe COVID-19 outcomes. By leveraging text network analysis, this study identified key research themes and their interconnections, offering valuable insights for future public health interventions and policymaking. Unlike traditional epidemiological models that analyze individual risk factors separately, our findings highlight the need for interdisciplinary approaches that integrate biomedical, social, and behavioral determinants to effectively mitigate severe COVID-19 outcomes.

### 4.4. Temporal Trends

Temporal analysis divided research trends into four phases:

Phase 1 (February–June 2020): Focused on early pandemic responses, including social distancing and transmission pathways [26].

Phase 2 (July–December 2020): As the pandemic progressed, research priorities shifted toward understanding population-specific vulnerabilities, including racial and socioeconomic disparities in infection rates and disease severity. During this period, the prolonged impact of lockdowns and healthcare system strain also contributed to increased interest in mental health challenges associated with pandemic stressors [27].

Phase 3 (January–March 2021): With the emergence of long-term effects of COVID-19, studies began to explore long COVID symptoms, post-viral syndromes, and their sociodemographic correlations [28].

Phase 4 (April–September 2021): Research efforts were increasingly directed toward vaccine development, public vaccination strategies, and the impact of emerging viral variants [29].

This progression reflects the evolving nature of research priorities in response to pandemic challenges, transitioning from immediate containment strategies to long-term health consequences and public health preparedness.

### 4.5. Strengths and Limitations

A key strength of this study is the use of advanced text network analysis, enabling a comprehensive evaluation of large-scale textual data. This approach provided insights into both established and emerging trends, supporting data-driven decision-making.

However, several limitations should be acknowledged. This study relied exclusively on English-language publications, introducing selection bias and limiting generalizability. The exclusion of non-peer-reviewed studies may have resulted in publication bias, omitting relevant but unpublished findings. Additionally, text network analysis has inherent limitations, including potential keyword bias, as term selection influences network structure. Temporal bias may also exist due to publication delays, potentially underrepresenting emerging risk factors. Furthermore, this study primarily utilized keyword frequency analysis and network-based methods, which identify associations between risk factors and severe COVID-19 outcomes but do not establish causal relationships. While the network analysis provides valuable insights into relational structures and co-occurrence patterns, it does not infer causality or determine the direct impact of specific risk factors. As a result, the findings should be interpreted as indicative of correlations rather than definitive causal links. To enhance the robustness of future research, additional statistical validation techniques (e.g., regression analysis, time-series modeling) and epidemiological modeling approaches (e.g., cohort studies, causal inference models, structural equation modeling) should be incorporated. These methods would help differentiate associative relationships from causal mechanisms, providing stronger empirical evidence for risk factor significance.

### 4.6. Implications and Future Directions

Findings from this study emphasize the need for interdisciplinary approaches to COVID-19 risk factors. While this study highlights key research themes, it is essential to translate these insights into clear and actionable public health strategies to improve pandemic preparedness.

Although COVID-19 is no longer a newly emerging crisis, its long-term effects—including post-infection complications, evolving variants, and pandemic-induced health disparities—continue to impact healthcare systems worldwide. The need to manage long COVID, chronic disease exacerbation, and the integration of pandemic lessons into future outbreak preparedness underscores the relevance of continued research in this field.

One of the critical aspects of pandemic response is ensuring that high-risk populations receive targeted interventions. Older adults, individuals with hypertension, and those with metabolic syndrome are particularly vulnerable to severe outcomes, necessitating tailored preventive measures and medical management strategies. In addition, efforts to expand healthcare access are crucial in mitigating disparities faced by vulnerable demographic groups who often experience systemic barriers to medical care.

Guidance on the appropriate use of non-invasive ventilation (NIV) is another area that requires refinement. Evidence-based guidelines should be established to ensure that NIV is utilized effectively, minimizing unnecessary intubations while optimizing respiratory support. Similarly, the psychological effects of lockdowns, economic distress, and post-infection trauma must be addressed through stronger mental health support systems integrated into pandemic response efforts.

Vaccine acceptance remains a major challenge in public health. Targeted communication strategies that address misinformation and vaccine hesitancy are essential in ensuring widespread immunization coverage. Effective campaigns should focus on culturally appropriate messaging and trust-building within communities.

Beyond these immediate public health measures, future research should incorporate statistical and epidemiological methodologies to strengthen the validity of findings derived from text network analysis. The application of regression models, time-series analysis, and epidemiological modeling—such as cohort studies, causal inference models, and structural equation modeling—would enhance causal inference and improve the robustness of risk factor identification.

As pandemics continue to evolve, integrating diverse analytical approaches will be crucial in shaping effective public health strategies. This study provides a comprehensive analytical framework that can be adapted for future infectious disease outbreaks, reinforcing data-driven decision-making in global health policy.

## 5. Conclusions

This study demonstrates the utility of the text network analysis in mapping knowledge structures and identifying trends in severe COVID-19 risk factors. The insights gained can inform targeted public health interventions, support data-driven policy development, and enhance pandemic preparedness. Specifically, our findings can help policymakers develop risk stratification models to identify high-risk populations, optimize vaccine distribution strategies, and improve early warning systems for emerging infectious diseases. Furthermore, the application of the text network analysis extends beyond COVID-19, offering a scalable approach for analyzing epidemiological data in future outbreaks. By leveraging such data-driven methodologies, decision-makers can implement more effective and proactive health strategies.

## Figures and Tables

**Table 1:** Keywords list with the highest appearance frequency.

Rank	Keyword	Frequency	PageRank Centrality	Rank	Frequency	Frequency	Page Rank Centrality
1	Age	3224	0.001136	26	Multiorgan failure	234	0.000272
2	Treatment	2934	0.000911	27	Asymptomatic	229	0.000576
3	Diabetes	1504	0.001191	28	Vitamin D	214	0.000961
4	Hypertension	1230	0.001232	29	Virus transmission	189	0.000343
5	Obesity	1045	0.000732	30	Anticoagulation	180	0.000866
6	Healthcare workers	960	0.00062	31	Mild cases	165	0.000211
7	Sex	941	0.000576	32	America	160	0.000569
8	Mean age	767	0.000665	33	COVID-19 treatment	152	0.000281
9	C-reactive protein	596	0.000865	34	Convalescent plasma	148	0.001066
10	Mental health	543	0.000887	35	IgM	142	0.001007
11	Social distancing	449	0.000203	36	Platelet	142	0.000565
12	Cardiovascular disease	428	0.000686	37	Nasopharyngeal swab	140	0.000631
13	IgG	428	0.00143	38	Cardiovascular system	134	0.000943
14	IL-6	415	0.000961	39	Immunosuppressant	133	0.000421
15	Cytokine storm	398	0.000573	40	Coronary heart disease	132	0.000446
16	COVID-19 vaccine	360	0.001217	41	Hyperglycemia	128	0.001327
17	Invasive mechanical ventilation	355	0.000295	42	Reverse transcription PCR	126	0.000377
18	Dyspnea	322	0.000465	43	Rheumatic disease	126	0.000924
19	COVID-19 transmission	319	0.000403	44	Non-invasive ventilation	124	0.001531
20	COVID-19 vaccination	319	0.000911	45	Glucocorticoid	116	0.00092
21	Lactate dehydrogenase	285	0.001003	46	Endotracheal intubation	115	0.000567
22	Acute kidney injury	273	0.000776	47	Chloroquine	113	0.00066
23	Tocilizumab	258	0.000549	48	Cancer care	108	0.000854
24	Remdesivir	257	0.001012	49	Vitamin D deficiency	108	0.000574
25	Heart failure	235	0.000947	50	Aerosol-generating procedures	107	0.001079

**Table 2:** Main keywords list of groups.

Group	Factors	Subtopic	Keywords
G1	Biomedicine factors	-	thrombolysis, acute pulmonary embolism, hyperglycemia, rheumatoid arthritis, kidney transplant, hemostasis, insulin resistance, transplant, major bleeding, liver transplant recipients, coagulopathy, low-molecular-weight heparin, coagulation abnormalities, glucose, transplant recipients, COVID-19-associated coagulopathy, rheumatic disease
G2	Occupational and environmental factors	-	oral cavity, surgical masks, personal protective equipment use, staff members, dental practice, mouth, nasal, residents, aerosol-generating procedures, dentist, otolaryngologist, long-term care facilities, nursing home, nursing home residents
G3	Demographic factors	-	non-hispanic black, racial disparities, white, black race, Latino, hypertension, diabetes, age, hospital, mortality, COVID-19 infection, COVID-19 patient, COVID-19 pandemic, health equity, obesity, mean age, sex
G4	Health behavior factors	-	Moderna, vaccine hesitancy, vaccine acceptance, tobacco smoking, mRNA vaccine, COVID-19 vaccine, allergic reactions, smoking cessation, epitope, cigarette smoking, nicotine, COPD patients, immunogenicity, current smokers, lung function, vaccine safety, alcohol, alcohol consumption
G5	Complication factors	C1 (Immunologic)	tumor necrosis factor, coagulation system, hyperinflammatory state, IgG, macrophage, IL-1, IL-10, NK cells, IL-2, IL-6, B cells, cytokine storm
		C2 (Psychiatric)	Patient Health Questionnaire-9, Insomnia Severity Index, poverty, mental health symptoms, insomnia symptoms, sleep quality, psychological symptoms, generalized anxiety, psychological status, mental health issues
		C3 (Respiratory)	respiratory status, non-invasive ventilation, nasal cannula, room air, prone positioning, bronchoscopy, high-flow nasal cannula
		C4 (Cardiac)	cardiac biomarkers, acute coronary syndrome, acute myocardial injury, cardiac arrhythmias, heart failure, pre-existing cardiovascular disease, CK-MB
		C5 (Neurologic)	encephalitis, Guillain-Barre syndrome, seizure, neurological disorders, neurological symptoms
		C6 (Gynecologic)	cesarean section, neonatal outcomes, breast milk, preterm birth, adverse pregnancy outcomes, breastfeeding, maternal mortality
		C7 (Secondary Infection)	Candida, bacteria, Klebsiella, Staphylococcus, bacterial infections, coinfection

**Table 3:** The appearance frequencies of the keywords in each phase.

Keywords	Phase 1	Phase 2	Phase 3	Phase 4
Obesity	91	320	170	464
Social distancing	67	144	62	176
Dyspnea	42	100	56	124
COVID-19 transmission	39	83	60	137
Chloroquine	32	41	15	25
Emergency department	30	101	63	159
America	27	55	28	50
Radiotherapy	26	24	11	21
Virus transmission	22	60	35	72
Coronary heart disease	22	34	25	51
Cancer care	21	39	16	32
Dentist	21	28	9	26
Perceived stress	-	13	15	34
Psychological symptoms	-	13	12	37
Hispanics	-	17	16	26
Generalized anxiety	-	15	10	24
Psychological well-being	-	10	8	30
Health disparities	-	15	11	13
Urea	-	15	9	14
Common complication	-	14	9	15
Deep venous thrombosis	-	13	10	11
Black race	1	21	9	26
Critical disease	1	20	14	26
Second wave	1	19	29	90
Persistent symptoms	-	2	10	22
Long COVID	-	-	6	21
Nationality	-	-	6	14
Mucormycosis	-	1	6	32
Moderna	-	1	5	44
First lockdown	-	1	5	21
Risk and protective factors	-	2	7	19
Anaphylaxis	-	2	6	27
Blood parameters	1	1	9	12
Sociodemographic data	-	2	8	10
mRNA vaccine	-	1	4	63
Delta	-	1	3	55
Bamlanivimab	-	-	-	35
Vaccine effectiveness	-	2	2	27
New variants	-	-	3	26
Vaccination coverage	-	-	3	23
Vaccine acceptance	-	1	3	19
Vaccination campaign	-	1	3	18
Kidney replacement therapy	1	3	-	17
Fungal infections	1	2	2	16

## Data Availability

The data from this study are available upon reasonable request from the corresponding author.

## Abbreviations

The following abbreviations are used in this manuscript:

CK-MB	Creatine Kinase-MB
COPD	Chronic Obstructive Pulmonary Disease
COVID-19	Coronavirus Disease 2019
IgG	Immunoglobulin G
IgM	Immunoglobulin M
IL-6	Interleukin-6
NIV	Non-Invasive Ventilation
SARS-CoV-2	Severe Acute Respiratory Syndrome Coronavirus 2
TF-IDF	Term Frequency–Inverse Document Frequency

## Appendix A

**Table A1:** Literature search results.

Database	Query Detailed	Number of Literature
PubMed	((COVID-19 OR SARS-CoV-2 OR 2019-nCoV OR 2019-Novel Coronavirus OR COVID OR COVID-2019 OR coronavirus-disease-2019 OR severe-acute-respiratory-syndrome-coronavirus-2 [Title/Abstract]) AND (risk[Title/Abstract] OR risk factor[Title/Abstract] OR predict[Title/Abstract]) AND (severe[Title/Abstract] OR critical[Title/Abstract] OR ICU care[Title/Abstract] OR mechanical ventilation[Title/Abstract] OR intensive care unit[Title/Abstract] OR severity[Title/Abstract] OR respiratory failure[Title/Abstract] OR fatal[Title/Abstract]))	17,674
CINAHL	(COVID-19 OR SARS-CoV-2 OR 2019-nCoV OR 2019-Novel Coronavirus OR COVID OR COVID-2019 OR coronavirus disease-2019 OR severe-acute-respiratory-syndrome-coronavirus-2) AND (risk OR risk factor OR predict) AND (severe OR critical OR ICU care OR mechanical ventilation OR intensive care unit OR severity OR respiratory failure OR fatal)	4367
EMBASE	‘coronavirus disease 2019′:ab,ti AND (‘risk factor’:ab,ti OR risk:ab,ti) AND (‘disease severity’:ab,ti OR ‘intensive care’:ab,ti OR ‘intensive care unit’:ab,ti OR ‘artificial ventilation’:ab,ti OR ‘respiratory failure’:ab,ti OR fatality:ab,ti)	1886
WoS	(COVID-19 OR SARS-CoV-2 OR 2019-nCoV OR 2019-Novel Coronavirus OR COVID OR COVID-2019 OR coronavirus disease-2019 OR severe-acute-respiratory-syndrome-coronavirus-2) AND (risk OR risk factor OR predict) AND (severe OR critical OR ICU care OR mechanical ventilation OR intensive care unit OR severity OR respiratory failure OR fatal)	18,973

**Table A2:** Keywords with the highest frequency of appearance.

Rank	Keyword	Frequency	PageRank Centrality	Rank	Keyword	Frequency	PageRank Centrality
1	Age	3224	0.001136	26	Multiorgan failure	234	0.000272
2	Treatment	2934	0.000911	27	Asymptomatic	229	0.000576
3	Diabetes	1504	0.001191	28	Vitamin D	214	0.000961
4	Hypertension	1230	0.001232	29	Virus transmission	189	0.000343
5	Obesity	1045	0.000732	30	Anticoagulation	180	0.000866
6	Healthcare workers	960	0.00062	31	Mild cases	165	0.000211
7	Sex	941	0.000576	32	America	160	0.000569
8	Mean age	767	0.000665	33	COVID-19 treatment	152	0.000281
9	C-reactive protein	596	0.000865	34	Convalescent plasma	148	0.001066
10	Mental health	543	0.000887	35	IgM	142	0.001007
11	Social distancing	449	0.000203	36	Platelet	142	0.000565
12	Cardiovascular disease	428	0.000686	37	Nasopharyngeal swab	140	0.000631
13	IgG	428	0.00143	38	Cardiovascular system	134	0.000943
14	IL-6	415	0.000961	39	Immunosuppressant	133	0.000421
15	Cytokine storm	398	0.000573	40	Coronary heart disease	132	0.000446
16	COVID-19 vaccine	360	0.001217	41	Hyperglycemia	128	0.001327
17	Invasive mechanical ventilation	355	0.000295	42	Reverse transcription PCR	126	0.000377
18	Dyspnea	322	0.000465	43	Rheumatic disease	126	0.000924
19	COVID-19 transmission	319	0.000403	44	Non-invasive ventilation	124	0.001531
20	COVID-19 vaccination	319	0.000911	45	Glucocorticoid	116	0.00092
21	Lactate dehydrogenase	285	0.001003	46	Endotracheal intubation	115	0.000567
22	Acute kidney injury	273	0.000776	47	Chloroquine	113	0.00066
23	Tocilizumab	258	0.000549	48	Cancer care	108	0.000854
24	Remdesivir	257	0.001012	49	Vitamin D deficiency	108	0.000574
25	Heart failure	235	0.000947	50	Aerosol-generating procedures	107	0.001079
51	CT scan	107	0.000351	76	chest CT	84	0.001138
52	preterm birth	107	0.001283	77	dentist	84	0.001023
53	physical activity	104	0.000525	78	healthy controls	84	0.000648
54	Australian	103	0.000668	79	myocardial infarction	84	0.000427
55	prone positioning	102	0.001243	80	laboratory markers	83	0.000251
56	chronic lung disease	100	0.000206	81	patient health questionnaire-9	82	0.002146
57	secondary infection	100	0.000857	82	post-traumatic stress disorder	82	0.000686
58	RT-PCR testing	99	0.00031	83	radiotherapy	82	0.000903
59	females	98	0.000337	84	antibody response	81	0.001636
60	healthy individuals	97	0.000384	85	coagulopathy	81	0.001146
61	sociodemographic factors	97	0.000348	86	European society	81	0.001098
62	hemoglobin	96	0.000811	87	renal failure	81	0.000399
63	hemodialysis	95	0.000422	88	viral pneumonia	81	0.00028
64	white blood cell count	95	0.001327	89	mechanically ventilated patients	80	0.000509
65	neutrophil count	93	0.001072	90	pregnant patients	80	0.000871
66	multisystem inflammatory syndrome	92	0.000643	91	antiviral therapy	79	0.000343
67	socioeconomic status	92	0.000641	92	inflammatory bowel diseases	79	0.000647
68	symptomatic COVID-19	92	0.000565	93	HIV infection	78	0.000598
69	corticosteroids	90	0.000509	94	cesarean section	77	0.001697
70	Switzerland	88	0.000393	95	innate immunity	77	0.000648
71	surgeon	86	0.001447	96	propensity score	77	0.000636
72	beneficial effect	85	0.000455	97	virus infection	77	0.000639
73	elective surgery	85	0.00102	98	endothelial cells	76	0.000272
74	receiver operating characteristic curve	85	0.000768	99	most common symptom	76	0.000351
75	autoimmune disease	84	0.000616	100	vasopressor	76	0.000295
101	white	76	0.001528	126	demographic information	65	0.000384
102	renin-angiotensin system	75	0.00149	127	Denmark	65	0.000808
103	standard care	74	0.001171	128	herd immunity	65	0.000386
104	lung disease	73	0.000365	129	immunomodulators	65	0.000949
105	SARS-CoV-2 spike protein	73	0.00097	130	neutrophil-to-lymphocyte ratio	65	0.000509
106	ventilatory support	73	0.00032	131	alcohol consumption	64	0.001007
107	chest radiography	72	0.000638	132	chronic liver disease	64	0.000581
108	SARS-CoV-2 pneumonia	72	0.000259	133	non-pharmaceutical interventions	64	0.000854
109	airway	69	0.000514	134	smoking status	64	0.000591
110	D-dimer level	69	0.000725	135	anti-SARS-CoV-2 antibodies	63	0.000759
111	thromboembolic complications	69	0.000557	136	atrial fibrillation	63	0.00037
112	ACE2 receptor	68	0.000268	137	belief	63	0.001276
113	genome	68	0.00076	138	California	63	0.000343
114	long-term care facilities	68	0.000932	139	contact tracing	63	0.000564
115	mRNA vaccine	68	0.001323	140	occupation	63	0.001077
116	worry	68	0.000817	141	oxygen support	63	0.000319
117	hematological malignancies	67	0.001023	142	self-isolation	63	0.000349
118	influenza vaccination	67	0.000821	143	transmissibility	63	0.000271
119	patient health	67	0.001849	144	central nervous system	62	0.001034
120	ACE-2	66	0.001213	145	methylprednisolone	62	0.000563
121	Bangladesh	66	0.00029	146	oxidative stress	62	0.000713
122	blood pressure	66	0.001118	147	perceived stress	62	0.000861
123	confidence	66	0.000745	148	psychological symptoms	62	0.001323
124	abdominal pain	65	0.000988	149	relapse	62	0.000413
125	anticoagulation therapy	65	0.001079	150	seroconversion	62	0.00123
151	critical disease	61	0.000496	176	Hispanics	59	0.001094
152	dysregulation	61	0.00051	177	IL-1	59	0.001205
153	IgA	61	0.000667	178	investigator	59	0.000704
154	insomnia severity index	61	0.001768	179	machine learning model	59	0.000408
155	insulin	61	0.001046	180	perceived risk	59	0.000321
156	mental health outcomes	61	0.000682	181	sequential organ failure assessment	59	0.001008
157	metabolism	61	0.000494	182	supplementation	59	0.001031
158	mobility	61	0.001083	183	acute coronary syndrome	58	0.001673
159	tobacco smoking	61	0.001362	184	chest pain	58	0.000251
160	anorexia	60	0.001089	185	conflict	58	0.000566
161	fibrosis	60	0.00037	186	immune function	58	0.000664
162	home quarantine	60	0.00043	187	preventive strategies	58	0.000322
163	hypoxemic respiratory failure	60	0.000434	188	prothrombin time	58	0.001617
164	lymphocytopenia	60	0.000542	189	rituximab	58	0.001095
165	males	60	0.00028	190	TNF-alpha	58	0.00094
166	neutralizing antibodies	60	0.000465	191	travel	58	0.000554
167	nosocomial transmission	60	0.000269	192	water	58	0.000605
168	rheumatoid arthritis	60	0.001324	193	alcohol	57	0.001026
169	vaccines	60	0.000779	194	bacteria	57	0.001565
170	zinc	60	0.001032	195	black race	57	0.001431
171	aerosolization	59	0.001502	196	immune dysregulation	57	0.000511
172	bed	59	0.0005	197	kidney transplant	57	0.001297
173	Delta	59	0.00024	198	lesion	57	0.00084
174	feeling	59	0.000414	199	positive test results	57	0.000272
175	healthcare resources	59	0.000209	200	social determinants	57	0.000525

**Table A3:** Jaccard similarity values by month.

Phase	Month	Similarity
1	2020.02.∼2020.03.	0.187
	2020.03.∼2020.04.	0.244
	2020.04.∼2020.05.	0.429
	2020.05.∼2020.06.	0.676
	2020.06.∼2020.07.	0.637
2	2020.07.∼2020.08.	0.660
	2020.08.∼2020.09.	0.650
	2020.09.∼2020.10.	0.695
	2020.10.∼2020.11.	0.662
	2020.11.∼2020.12.	0.676
	2020.12.∼2021.01.	0.603
3	2021.01.∼2021.02.	0.697
	2021.02.∼2021.03.	0.677
	2021.03.∼2021.04.	0.655
4	2021.04.∼2021.05.	0.679
	2021.05.∼2021.06.	0.695
	2021.06.∼2021.07.	0.698
	2021.07.∼2021.08.	0.690
	2021.08.∼2021.09.	0.697
	2021.09.∼2021.10.	0.687

## References

[1] World Health Organization (2020). Coronavirus Disease (COVID-19) Pandemic. https://www.who.int/emergencies/diseases/novel-coronavirus-2019.

[2] Centers for Disease Control and Prevention COVID-19 Overview and Infection Prevention and Control Priorities in Non-U.S. Healthcare Settings.. https://archive.cdc.gov/www_cdc_gov/coronavirus/2019-ncov/hcp/non-us-settings/overview/index.html.

[3] Dong E., Du H., Gardner L. (2020). An interactive web-based dashboard to track COVID-19 in real time. Lancet Infect. Dis..

[4] Zhou F., Yu T., Du R., Fan G., Liu Y., Liu Z., Xiang J., Wang Y., Song B., Gu X. (2020). Clinical course and risk factors for mortality of adult inpatients with COVID-19 in Wuhan, China: A retrospective cohort study. Lancet.

[5] Wang C., Horby P.W., Hayden F.G., Gao G.F. (2020). A novel coronavirus outbreak of global health concern. Lancet.

[6] Eysenbach G. (2020). How to fight an infodemic: The four pillars of infodemic management. J. Med. Internet Res..

[7] Lodhwal V., Choudhary G. (2023). Survey Paper: Automatic Title Generation for Text with RNN and Pre-trained Transformer Language Model. Int. J. Res. Appl. Sci. Eng. Technol..

[8] Dritsas E., Trigka M. (2025). Exploring the intersection of machine learning and big data: A survey. Mach. Learn. Knowl. Extr..

[9] Park S., Park J. (2021). Identifying the knowledge structure and trends of outreach in public health care: A text network analysis and topic modeling. Int. J. Environ. Res. Public Health.

[10] Kim H.J., Bae S.H., Park J.H. (2023). Research trends on cancer-related cognitive impairment in patients with non-central nervous system cancer: Text network analysis and topic modeling. J. Korean Acad. Fundam. Nurs..

[11] Lee S.S. (2013). Analytical Study on the Relationship between Centralities of Research Networks and Research Performances. J. Korean Libr. Inf. Sci. Soc..

[12] Zhang H., Wu Y., He Y., Liu X., Liu M., Tang Y., Xu S., Wang M., Wang W., Liang G. (2022). Age-related risk factors and complications of patients with COVID-19: A population-based retrospective study. Front. Med..

[13] Du Y., Zhou N., Zha W., Lv Y. (2021). Hypertension is a clinically important risk factor for critical illness and mortality in COVID-19: A meta-analysis. Nutr. Metab. Cardiovasc. Dis..

[14] Fan X., Han J., Zhao E., Fang J., Wang D., Cheng Y., Shi Y., Wang Z., Yao Z., Lu P. (2023). The effects of obesity and metabolic abnormalities on severe COVID-19-related outcomes after vaccination: A population-based study. Cell Metab..

[15] Wu S., Zhou K., Misra-Hebert A., Bena J. (2022). Impact of metabolic syndrome on severity of COVID-19 illness. Metab. Syndr. Relat. Disord..

[16] Ramirez G.A., Bozzolo E.P., Gobbi A., Castelli E., Centurioni C., Di Meo M., Della Torre E., Di Scala F., Morgillo A., Marinosci A. (2022). Outcomes of noninvasive ventilation as the ceiling of treatment in patients with COVID-19. Panminerva Med..

[17] Castro R.A., Frishman W.H. (2021). Thrombotic complications of COVID-19 infection: A review. Cardiol. Rev..

[18] Galanis P., Vraka I., Fragkou D., Bilali A., Kaitelidou D. (2021). Impact of personal protective equipment use on health care workers’ physical health during the COVID-19 pandemic: A systematic review and meta-analysis. Am. J. Infect. Control.

[19] Mackey K., Ayers C.K., Kondo K.K., Saha S., Advani S.M., Young S., Spencer H., Rusek M., Anderson J., Veazie S. (2021). Racial and ethnic disparities in COVID-19-related infections, hospitalizations, and deaths: A systematic review. Ann. Intern. Med..

[20] Schoch-Spana M., Brunson E.K., Long R., Ruth A., Ravi S.J., Trotochaud M., Borio L., Brewer J., Buccina J., Connell N. (2021). The public’s role in COVID-19 vaccination: Human-centered recommendations to enhance pandemic vaccine awareness, access, and acceptance in the United States. Vaccine.

[21] Mohammed I., Nauman A., Paul P., Ganesan S., Chen K.-H., Jalil S.M.S., Jaouni S.H., Kawas H., Khan W.A., Vattoth A.L. (2022). The efficacy and effectiveness of the COVID-19 vaccines in reducing infection, severity, hospitalization, and mortality: A systematic review. Hum. Vaccin. Immunother..

[22] Chodkiewicz J., Talarowska M., Miniszewska J., Nawrocka N., Bilinski P. (2020). Alcohol consumption reported during the COVID-19 pandemic: The initial stage. Int. J. Environ. Res. Public Health.

[23] Guan W.-J., Ni Z.-Y., Hu Y., Liang W.-H., Ou C.-Q., He J.-X., Liu L., Shan H., Lei C.-L., Hui D.S.C. (2020). Clinical characteristics of coronavirus disease 2019 in China. N. Engl. J. Med..

[24] Liu R., Han H., Liu F., Lv Z., Wu K., Liu Y., Feng Y., Zhu C. (2020). Positive rate of RT-PCR detection of SARS-CoV-2 infection in 4880 cases from one hospital in Wuhan, China, from Jan to Feb 2020. Clin. Chim. Acta.

[25] Zhong R., Chen L., Zhang Q., Li B., Qiu Y., Wang W., Tan D., Zou Y. (2021). Which factors—Smoking, drinking alcohol, betel quid chewing, or underlying diseases—Are more likely to influence the severity of COVID-19?. Front. Physiol..

[26] Zhang L., Zhao W., Sun B., Huang Y., Glänzel W. (2020). How scientific research reacts to international public health emergencies: A global analysis of response patterns. Scientometrics.

[27] Thomeer M.B., Moody M.D., Yahirun J. (2023). Racial and ethnic disparities in mental health and mental health care during the COVID-19 pandemic. J. Racial Ethn. Health Disparities.

[28] Crook H., Raza S., Nowell J., Young M., Edison P. (2021). Long covid—Mechanisms, risk factors, and management. BMJ.

[29] Polack F.P., Thomas S.J., Kitchin N., Absalon J., Gurtman A., Lockhart S., Perez J.L., Pérez Marc G., Moreira E.D., Zerbini C. (2020). Safety and efficacy of the BNT162b2 mRNA COVID-19 vaccine. N. Engl. J. Med..

